# Specific membrane capacitance, cytoplasm conductivity and instantaneous Young’s modulus of single tumour cells

**DOI:** 10.1038/sdata.2017.15

**Published:** 2017-02-14

**Authors:** Ke Wang, Yang Zhao, Deyong Chen, Beiyuan Fan, Yulan Lu, Lianhong Chen, Rong Long, Junbo Wang, Jian Chen

**Affiliations:** 1 State Key Laboratory of Transducer Technology, Institute of Electronics, Chinese Academy of Sciences, Beijing 100190, PR China; 2 School of Electronic, Electrical and Communication Engineering, University of Chinese Academy of Sciences, Beijing 100190, PR China; 3 R&D Center for Healthcare Electronics, Institute of Microelectronics, Chinese Academy of Sciences, Beijing 100029, PR China; 4 Department of Mechanical Engineering, University of Colorado, Boulder, Colorado 80309, USA

**Keywords:** Nanoscale biophysics, Microfluidics, Lab-on-a-chip

## Abstract

As label-free biomarkers, biophysical properties of cells are widely used for cell type classification. However, intrinsic biophysical markers, e.g., specific membrane capacitance (C_specific membrane_), cytoplasm conductivity (σ_conductivity_) and instantaneous Young’s modulus (E_instantaneous_) measured for hundreds of single cells were not yet reported. In this study, single cells in suspension (adherent cells treated with trypsin) were aspirated through a microfluidic constriction channel at 25 °C, and the entry processes and impedance profiles were recorded and translated to C_specific membrane_, σ_conductivity_ and E_instantaneous_. C_specific membrane_, σ_conductivity_ and E_instantaneous_ of five cell types were quantified as 2.10±0.38 μF cm^−2^, 0.91±0.15 S m^−1^ and 5.52±0.95 kPa for H460 cells (n_cell_=437); 2.52±0.54 μF cm^−2^, 0.83±0.12 S m^−1^ and 5.54±1.04 kPa for H446 cells (n_cell_=410); 2.45±0.57 μF cm^−2^, 0.99±0.18 S m^−1^ and 5.16±1.68 kPa for A549 cells (n_cell_=442); 1.86±0.31 μF cm^−2^, 1.07±0.18 S m^−1^ and 3.86±0.81 kPa for 95D cells (n_cell_=415); 2.03±0.35 μF cm^−2^, 0.99±0.16 S m^−1^ and 3.49±0.70 kPa for 95C cells (n_cell_=290). The database of C_specific membrane_, σ_conductivity_ and E_instantaneous_ may serve as a reference for future studies of cellular biophysical properties.

## Background & Summary

Biophysical properties of single cells include electrical parameters such as specific membrane capacitance (C_specific membrane_, i.e., capacitance per unit area for the cell membrane modelled as a capacitor) and cytoplasm conductivity (σ_conductivity_, i.e., the reciprocal of electrical resistivity or specific electrical resistance for the cytoplasm modelled as a resistor) as well as mechanical parameters such as instantaneous Young’s modulus (E_instantaneous_, which describes the initial elastic response of a cell, modelled as an incompressible viscoelastic solid, in response to mechanical forces), which are indicators of the status of cytoskeletons and cellular membranes^1^. Variations in cellular biophysical properties are closely related to physiological and pathological processes, examples including (1) red blood cells infected by malaria; (2) tumour cells during migration and evasion; (3) leukocytes affected by sepsis; and (4) stem cells undergoing differentiation^2,3^.

Conventional techniques for characterizing the electrical properties of cells include patch clamping, electrorotation and dielectrophoresis^4–7^. On the other hand, atomic force microscopy, micropipette aspiration and optical tweezers have been used for characterizing the mechanical properties of cells^8–12^. Although well established, these approaches suffer from limited throughput (~1 cell per minute). In addition, they are not capable of measuring cellular electrical and mechanical properties simultaneously^1^. Therefore, data recording C_specific membrane_, σ_conductivity_ and E_instantaneous_ for multiple cell types (hundreds of cells for each cell type) are currently not available in the literature.

Microfluidics is an area focusing on processing small amounts of fluids at the nanoliter to picoliter scale using microfabricated channels with dimensions of tens of micrometers^13–15^. The micrometer dimension is comparable to biological cells, and thus microfluidics is well suited for single-cell analysis^16,17^. More specifically, advances in microfluidic technology have enabled high-throughput characterization of the biophysical properties of single cells, e.g., through impedance flow cytometry, microfluidic optical stretchers and microfluidic hydrodynamic stretchers^1,8,18^. Although powerful, these previously reported microfluidics-based approaches only reported electrical (e.g., impedance flow cytometry) or mechanical (e.g., microfluidic optical stretchers and hydrodynamic stretchers) properties of single cells separately, without demonstrating simultaneous characterization of cellular electrical and mechanical properties.

Microfluidic approaches that enable simultaneous characterization of electrical and mechanical properties of single cells have also been developed, specifically through (1) microcantilever-based electrodes^19^, (2) electrodeformation^20^, (3) micropipette aspiration with impedance spectroscopy^21^, or (4) constriction channels with impedance spectroscopy^22^. The first three approaches are limited by their low throughput, and thus cannot be used to collect data from hundreds of single cells. In the fourth approach, cells in suspension were aspirated through a constriction channel (with a width and a height that are smaller than the cell diameter) continuously. The deformation and impedance profiles of individual cells as they enter and travel through the constriction channel were recorded as mechanical and electrical signals, respectively, with an estimated throughput of 1 cell per second.

Based on custom-developed electromechanical models, these raw biophysical signals obtained from constriction channels with impedance spectroscopy were then translated to size-independent intrinsic biophysical markers including C_specific membrane_, σ_conductivity_ and E_instantaneous_^23–25^, enabling the classification of tumour cells with different malignant levels^26^. However, as proof-of-concept demonstrations, only a limited number of cell types were examined with small populations for each cell type^24^.

In this study, based on the aforementioned approach, C_specific membrane_, σ_conductivity_ and E_instantaneous_ from five types of tumour cells and hundreds of single cells for each cell type were quantified and reported. This study provides a preliminary database for cellular C_specific membrane_, σ_conductivity_ and E_instantaneous_, which may serve as a reference for future studies on characterizing and classifying biological cells based on cellular biophysical properties.

## Methods

### Working flowchart

**Figure 1:**
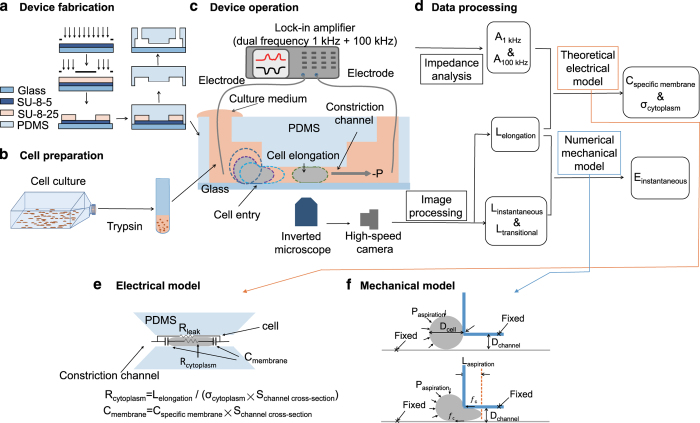
Working flowchart for simultaneous characterization of C_specific membrane_, σ_conductivity_ and E_instantaneous_ of single cells. Key steps include device fabrication (**a**), cell preparation (**b**), device operation (**c**) and data processing (**d**) leveraging developed electrical (**e**) and mechanical (**f**) models. During device operation, suspended cells were aspirated into the microfluidic constriction channels with the cell deformation and impedance profiles recorded by a high-speed camera and an impedance analyser, respectively. Preliminary biophysical markers including A_1 kHz_, A_100 kHz_, L_instantaneous_, L_transitional_ and L_elongation_ were obtained based on processing of image and impedance data, which were then translated to C_specific membrane_, σ_conductivity_ and E_instantaneous_, based on a theoretical electrical model for cells traveling within the constriction channel and a numerical mechanical model capturing cell deformation during the entry process, respectively.

The working flowchart for characterizing C_specific membrane_, σ_conductivity_ and E_instantaneous_ includes four key steps: device fabrication, cell preparation, device operation and data processing (see Fig. 1). During operation, cells in suspension were aspirated into the microfluidic constriction channels with the deformation and impedance profiles of the cells recorded by a high-speed camera and an impedance analyser, respectively. Raw biophysical data were obtained by processing the images and impedance data captured in experiments, which were then translated to C_specific membrane_, σ_conductivity_ and E_instantaneous_, based on a theoretical electrical model for a cell traveling within the constriction channel and a numerical mechanical model capturing the deformation of a cell when it enters the constriction channel, respectively. The key steps were summarized as follows. The detailed procedures have been reported in a previous publication^24^.

### Device fabrication

The microfluidic device consists of a constriction channel (a cross sectional area of 10 μm×10 μm) in polydimethylsiloxane (PDMS) elastomer (Dow Corning Corp., Midland, MI, USA) which was replicated from a SU-8 (MicroChem Corp., Newton, MA, USA) mould master (see Fig. 1a).

Briefly, SU-8 5 was spun coated, prebaked and exposed without development and post exposure bake to form the layer of the constriction channel (10 μm in height). Then SU-8 25 was spin coated on top of the first SU-8 layer, exposed with alignment and developed, forming the cell loading channel with a height of 25 μm. PDMS precursor and curing agents (10:1 in weight) were mixed, poured on channel masters and baked for crosslinking. PDMS channels were then peeled away from the SU-8 masters, punched to form through holes as inlets and outlets, and bonded to glass slides after plasma treatment.

### Cell preparation

All cell-culture reagents were purchased from Life Technologies Corporation (Carlsbad, CA, USA). The lung cancer cell lines of H460, H446, A549, 95D and 95C (China Infrastructure of Cell Line Resources, Beijing, China) were cultured at 37 °C in 5% CO_2_ in RPMI 1640 medium (11875) supplemented with 10% heat-inactivated fetal bovine serum (10099), 100 units ml^−1^ penicillin and 100 μg ml^−1^ streptomycin (15140). Immediately prior to an experiment, cells were trypsinized (25200, 0.25% for 3 min) to form a solution of cell suspension at a concentration of 1×10^6^ cells ml^−1^ (see Fig. 1b).

### Device operation

During operation the device was first filled with culture medium and and a pipette was used to transfer the cell suspension solution to the entrance of the cell loading channel. A pressure calibrator (DPI-610 pressure calibrator, Druck, Billerica, MA, USA) was used to generate a negative pressure (0.5–1.2 kPa), aspirating cells continuously through the constriction channel with silver wires inserted into the inlet and the outlet of the device for impedance profile recording (see Fig. 1c).

An inverted microscope (IX71, Olympus Inc., Tokyo, Japan) in connection with a high-speed camera (M320S, Phantom Inc., Bublin, OH, USA) was used to capture the process of cellular entry and travelling in the constriction channel at 200 frames per second. Impedance data at both 1 and 100 kHz was measured by a lock-in amplifier (7270, Signal Recovery, Oak Ridge, TN, USA) with a sampling rate of 25 points per second. All the characterization experiments were conducted within 30 min trypsinization of the cells at the room temperature (25 °C).

### Data processing

At the stage when a cell enters the constriction channel, two preliminary parameters were quantified based on image processing: L_instantaneous_ as the aspiration length when a cell instantaneously jumps into the channel and L_transitional_ as the aspiration length at the point where the creep deformation of the cell ends (see Fig. 1d)^25^. During the stage when the cell is travelling within the constriction channel, the impedance profiles and images of the elongated cell were analysed which produced three preliminary parameters: A_1 kHz_ and A_100 kHz_, i.e., the ratios between the impedance amplitudes with and without a travelling cell at 1 and 100 kHz, respectively, as well as L_elongation_, i.e., aspiration length of the cell when travelling within the constriction channel (see Fig. 1d)^23^.

A theoretical model^23^ was previously developed to model the electrical response of a cell as it is traveling within the constriction channel, thereby enabling the conversion of the measured A_1 kHz_, A_100 kHz_ and L_elongation_ to C_specific membrane_ and σ_conductivity_ as size-independent intrinsic electrical markers for single cells. Briefly, the electrical response of a cell was represented by R_cytoplasm_ and C_membrane_ in series where R_leak_ indicated sealing properties during the cellular travelling process in the constriction channel. R_leak_ was derived from impedance data at 1 kHz (A_1 kHz_) and C_membrane_ and R_cytoplasm_ were derived from impedance at 100 kHz (A_100 kHz_), respectively, which, were further translated to C_specific membrane_ and σ_cytoplasm_ based on geometrical information of the constriction channel (see Fig. 1e). Note that in this study, the lumped electrical model was used for data interpretation where the membrane portion was represented by C_membrane_ and the cytoplasm portion was represented by R_cytoplasm_, which, to an extent, neglects the potential effects of ion channels in the cell membrane and the membranes of cytosolic organelles in cytoplasm. Future studies may develop more accurate cellular electrical models.

On the other hand, a numerical mechanical model^25^ was developed to model the cell deformation as it enters the constriction channel, where the channel walls were modelled as rigid surfaces and the cell was modelled as an incompressible viscoelastic solid. The key mechanical parameter to be extracted was E_instantaneous_. Based on numerical simulations, the relationships between L_instantaneous_, L_transitional_ and E_instantaneous_ were obtained as follows:

$$\begin{array}{l} L_{ins\tan\tan eous}/D_{channel}=\left(44.27f_{c}^{2}-37.24f_{c}+13.70\right)\times P_{aspiration}/E_{ins\tan\tan eous}+\left(-5.31f_{c}^{2}+2.84f_{c}-0.59\right)\\ L_{transitional}/D_{channel}=\left(-60.40f_{c}^{2}+40.05f_{c}-8.68\right)\times P_{aspiration}/E_{ins\tan\tan eous}+\left(1.99f_{c}^{2}+0.03f_{c}+1.60\right) \end{array}$$

where D_channel_ represents channel geometrical information, *ƒ*_c_ represents the friction on cell-wall interfaces and P_aspiration_ represents pressure applied to aspirate cells into the constriction channel (see Fig. 1f). The two unknown parameters, i.e., E_instantaneous_ and *ƒ*_c_, were solved from these two equations since all the other parameters can be measured from experiments.

## Data Records

Raw impedance and image data of five cell types are available at *Dryad repository* (Data Citation 1, Raw Impedance and Image Data of Single Tumour Cells). The file is an Excel composed of five sheets named after five cell lines of H460, H446, A549, 95D and 95C, respectively. In each sheet of a specific cell type, the first row is the cell number and within each cell number, there are seven columns which are time (collected from impedance analyser), amplitude at 1 kHz, phase at 1 kHz, amplitude at 100 kHz, phase at 100 kHz, time (collected from high-speed camera) and aspiration length.

C_specific membrane_, σ_conductivity_ and E_instantaneous_ of five cell types are available at *Dryad repository* (Data Citation 1, Biophysical Properties of Single Tumour Cells). The file is an Excel composed of five sheets named after five cell lines of H460, H446, A549, 95D and 95C, respectively. In each sheet of a specific cell type, there are nine columns which are cell number (column A), A_1 kHz_ (column B), A_100 kHz_ (column C), L_instantaneous_ (column D), L_transitional_ (column E), L_elongation_ (column F), C_specific membrane_ (column G), σ_conductivity_ (column H) and E_instantaneous_ (column I). In this file, each row represents the biophysical parameters of a specific cell, including A_1 kHz_, A_100 kHz_, L_instantaneous_, L_transitional_, L_elongation_, C_specific membrane_, σ_conductivity_ and E_instantaneous_.

## Technical Validation

**Figure 2:**
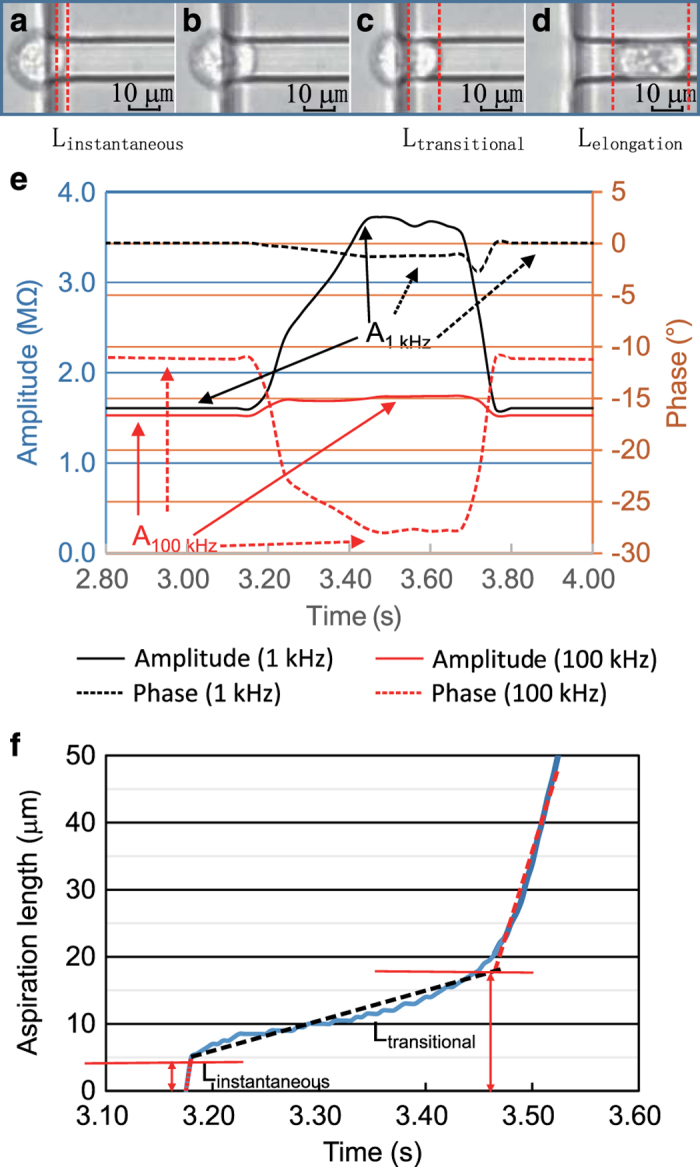
Raw imaging and impedance data for a typical cell to enter and travel in the constriction channel (length: 200 μm, width: 10 μm and height: 10 μm) with preliminary biophysical parameters quantified. (**a**–**d**) Microscopic pictures of a cell’s entry and travelling process in the constriction channel with raw impedance data of the same cell shown in **e** and processed aspiration length versus time shown in **f**. Based on data processing, five preliminary biophysical parameters including A_1 kHz_, A_100 kHz_, L_instantaneous_, L_transitional_, and L_elongation_ were obtained.

Figure 2 shows a sequence of microscopic images (a–d), showing the entry process into the constriction channel for a single cell. In addition, the impedance data for the same cell was shown in Fig. 2e and the aspiration length as a function of time was obtained by image processing and shown in Fig. 2f. The detailed data for individual cells are included in (Data Citation 1, Raw Impedance and Image Data of Single Tumour Cells).

Based on the processing of impedance profiles and image processing, five preliminary biophysical markers of A_1 kHz_, A_100 kHz_, L_instantaneous_, L_transitional_, and L_elongation_ were obtained. When the cell entered the constriction channel, an instantaneous jump into the constriction channel was initially observed (see Fig. 2a), enabling the quantification of L_instantaneous_ in Fig. 2e. Then, a gradual increase in aspiration length (see Fig. 2b,e) was observed due to viscoelastic creep. The creep deformation ended when L_transitional_ was reached (see Fig. 2c,e). Figure 2d shows an image of the cell travelling within the constriction channel where L_elongation_ was quantified. Furthermore, impedance ratios at A_1 kHz_ and A_100 kHz_ were also quantified during the cellular travelling process in the constriction channel (see Fig. 2e). The averages and standard deviations of five preliminary biophysical markers (A_1 kHz_, A_100 kHz_, L_instantaneous_, L_transitional_ and L_elongation_) for five types of tumour cells were summarized in Table 1. The detailed data for individual cells are included in (Data Citation 1, Biophysical Properties of Single Tumour Cells).

**Figure 3:**
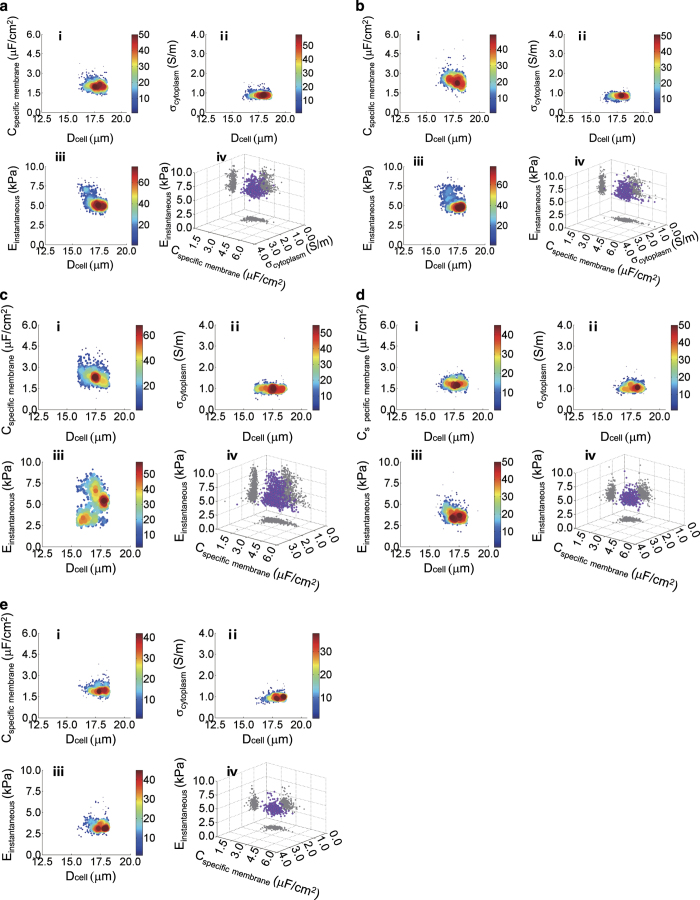
Scatter plots of C_specific membrane_, σ_conductivity_ and E_instantaneous_ of five tumour cells. The number of measured cells for each type was 437 for H460 (**a**), 410 for H446 (**b**), 442 for A549 (**c**), 415 for 95D (**d**) and 290 for 95C cells (**e**). In addition, for each cell type, scatter plots of (i) C_specific membrane_ versus D_cell_, (ii) σ_conductivity_ versus D_cell_, (iii) E_instantaneous_ versus D_cell_ and (iv) C_specific membrane_ versus σ_conductivity_ versus E_instantaneous_ were included.

Figure 3a–e show the distributions of C_specific membrane_, σ_conductivity_ and E_instantaneous_ for five types of tumour cells, respectively. For each type of tumour cells, the scatter plots of (i) C_specific membrane_ versus D_cell_, (ii) σ_conductivity_ versus D_cell_, (iii) E_instantaneous_ versus D_cell_ and (iv) C_specific membrane_ versus σ_conductivity_ versus E_instantaneous_ were included. Note that D_cell_ represents the diameter of the cell under measurement, which was calculated from L_elongation_ based on the assumption of volume conservation when the cell deforms. As shown in Table 1, C_specific membrane_, σ_conductivity_ and E_instantaneous_ of five cell types were found to be 2.10±0.38 μF cm^−2^, 0.91±0.15 S m^−1^ and 5.52±0.95 kPa for H460 cells (n_cell_=437); 2.52±0.54 μF cm^−2^, 0.83±0.12 S m^−1^ and 5.54±1.04 kPa for H446 cells (n_cell_=410); 2.45±0.57 μF cm^−2^, 0.99±0.18 S m^−1^ and 5.16±1.68 kPa for A549 cells (n_cell_=442); 1.86±0.31 μF cm^−2^, 1.07±0.18 S m^−1^ and 3.86±0.81 kPa for 95D cells (n_cell_=415); 2.03±0.35 μF cm^−2^, 0.99±0.16 S m^−1^ and 3.49±0.70 kPa for 95C cells (n_cell_=290). The detailed data of C_specific membrane_, σ_conductivity_ and E_instantaneous_ for individual cells are included in (Data Citation 1, Biophysical Properties of Single Tumour Cells).

## Additional Information

**How to cite this article:** Wang, K. *et al.* Specific membrane capacitance, cytoplasm conductivity and instantaneous Young’s modulus of single tumour cells. *Sci. Data* 4:170015 doi: 10.1038/sdata.2017.15 (2017).

**Publisher’s note:** Springer Nature remains neutral with regard to jurisdictional claims in published maps and institutional affiliations.

## Supplementary Material



## Figures and Tables

**Table 1:** A summary of biophysical parameters of five types of tumour cells.

**Cell Type**	**A**_**1 kHz**_	**A**_**100 kHz**_	**L**_**instantaneous**_ **(μm)**	**L**_**transitional**_ **(μm)**	**L**_**elongation**_ **(μm)**	**C**_**specific membrane**_ **(μF cm^−2^)**	**σ**_**cytoplasm**_ **(S m^−1^)**	**E**_**instantaneous**_ **(kPa)**
H460 (n_celll_=437)	3.41±0.47	1.17±0.02	8.99±1.15	18.14±2.63	28.18±2.42	2.10±0.38	0.91±0.15	5.52±0.95
H446 (n_celll_=410)	2.94±0.43	1.16±0.03	9.20±1.16	16.95±1.55	28.29±2.46	2.52±0.54	0.83±0.12	5.54±1.04
A549 (n_celll_=442)	3.14±0.56	1.14±0.03	8.21±1.48	17.31±2.57	27.59±3.66	2.45±0.57	0.99±0.18	5.16±1.68
95D (n_celll_=415)	3.49±0.49	1.17±0.03	8.56±1.01	17.68±1.80	27.86±2.34	1.86±0.31	1.07±0.18	3.86±0.81
95C (n_celll_=290)	3.33±0.43	1.19±0.03	9.38±1.21	19.06±2.09	28.64±2.31	2.03±0.35	0.99±0.16	3.49±0.70
Five preliminary biophysical parameters (e.g., A_1 kHz_, A_100 kHz_, L_instantaneous_, L_transitional_ and L_elongation_) and three size-independent intrinsic biophysical parameters (e.g., C_specific membrane_, σ_cytoplasm_ and E_instantaneous_) were included.								
